# A new charophyte habitat with a stabilized good ecological potential of mine water

**DOI:** 10.1038/s41598-021-93827-z

**Published:** 2021-07-15

**Authors:** Agnieszka Napiórkowska-Krzebietke, Andrzej Robert Skrzypczak

**Affiliations:** 1 Department of Ichthyology, Hydrobiology and Aquatic Ecology, S. Sakowicz Inland Fisheries Institute in Olsztyn, Olsztyn, Poland; 2 Institute of Engineering and Environmental Protection, Faculty of Geoengineering, University of Warmia and Mazury in Olsztyn, Olsztyn, Poland

**Keywords:** Ecology, Plant sciences, Ecology, Environmental sciences, Chemistry

## Abstract

Each newly-created pond which is supplied with mine water gives the opportunity to study a unique ecosystem in context of possible conditions for biotic live. Therefore, this research aimed to assess a phytoplankton-based ecological potential against the trophic conditions and the risk of contamination with trace elements, and demonstrate the possibility to stabilize at least good water quality of a clarification pond. The gradual decrease in turbidity-related variables (including suspended solids and iron) and nutrients, on the one hand, and an increase in phytoplankton-related indicators, on the other hand, were the most evident. Besides, relative stability in trace elements (the best water quality class), trophic level (slightly eutrophic level) and ecological potential (maximum potential), and relative instability in sulfates and calcium were also recorded. The final stabilization of water habitat resulted in abundant growth of charophyte *Nitella mucronata*. This all suggested a new ecological opportunity for settlement of a rare species and important for biodiversity enhancement. Furthermore, the study revealed that a clarification pond did not pose any toxic risk from the elevated content of trace elements or the growth of toxic or potentially toxic cyanobacteria which is essential for proper functioning and management of water ecosystems.

## Introduction

The newly human-formed water bodies serve a unique and added value to global aquatic ecosystems. Starting from the first years after fulfilling with water of different origin, they give an exceptional opportunity to study the stages of colonization by freshwater communities. They also play a very important role in the mining landscape fulfilling both the ecological and socio-economic functions. The typical mine water bodies include the so-called pit lakes which primarily are important for recreational purposes, fisheries, aquaculture, nature protected areas (new ecosystems subjected to biodiversity conservation plans), water management, flood protection and geochemical sink^1,2^. Recently, the creation of new so-called pit lakes flooding with the post-mining waters has become the most common and well known worldwide in thousands quantities^1,3^. In many cases, the mine water can constitute a real toxic or hazardous threat for ecosystem^4,5^. However, some ponds supplied with mine water can serve a new freshwater habitat characterized by a high biodiversity^6^.

Other aspects of a special concern should include the assessment of trophic state and ecological potential of various surface water bodies supplied with mine water which recently have been commonly established in Europe. For example, more than half thousand post-mining ponds and pit lakes were created in Germany^1^. Up to date, such water bodies were not subjected to common water policy for European countries, which since 2000 have been obligated to reduce pressures, achieve at least good ecological status for all water bodies and protect them^7^. The European Union Guidance Document on Eutrophication^8^ determines the “high” and “good” ecological status if phytoplankton growth does not or only in a slight degree cause the undesirable interferences to the water ecosystem. Such undesirable interferences can include persistent blooms especially due to the harmful cyanobacteria growth and consequently the macrophyte (including species of the genera *Chara*, *Nitella* and *Lychnothamnus*) loss. The most macrophytes, including charophytes, dominate at lower nutrient concentrations and develop in higher densities than most angiosperms^9^, and the charophyte abundance is usually negatively correlated with the concentration of ammonium at the bottom and dissolved fractions of mineral phosphorus (soluble reactive phosphorus SRP)^10^.

The present study focus on an artificial water body supplied with mine water. It fulfills the technical function as a clarification pond in pretreatment of water from dewatering system of the Bełchatów Coal Mine in Poland. The previous study initially confirmed that such water bodies can serve the good conditions for rich biological communities, primarily zooplankton and fish or even cannot be conducive to aquatic fauna growth^11–13^. Thus, it suggested a real possibility to establish the biological potential of such clarification ponds. The water bodies impacted by abandoned coal mines has recently become an interest of Environmental Agency in England^14^ due to the implementation of the Water Framework Directive (WFD) but mainly in the context of mine water discharges and the possible pollution.

The aims of the study were to (1) assess a phytoplankton-based ecological potential against the trophic conditions and contamination with trace elements, and (2) demonstrate the possibility to stabilize at least good water quality of a newly-formed clarification pond for a new habitat during the 6-year period. This research can be treated as an approach to further manage of the water resources including the newly formed water bodies not only in local or European scale but also in global scale regarding freshwater management, policy and conservation.

## Results

### Environmental variables, toxic risk and trophic conditions

During the 6-year period, the highest average temperature and oxygen saturation were noted in summer, while their lowest averages in winter (Supplementary Table S1). In summer, temperature ranged from 18.1 to 21.7 °C and oxygen content from 8.1 to 9.8 mg L^−1^. In winter, in turn, temperature and oxygen content ranged from 9.7 to 13.1 °C and from 8.6 to 11.8 mg L^−1^ respectively. Both the spring and autumn seasons were characterized by similar averages of water temperature and oxygen content. The thermal-oxygen conditions were not statistically different between the years (*P* > 0.05).

The water in the Kuźnica clarification pond was slightly alkaline with pH range of 7.4–8.4 (Supplementary Table S2). The SDD ranged from 0.42 to 0.69 m, on average, and it was statistically lower in 2014 than in 2018 and 2019 (*P* = 0.0003). Significant differences were observed in turbidity between two periods (*P* = 0.0003) with averages of 37.3–40.7 NTU and 11.8–14.0 NTU, respectively in 2014–2016 and 2017–2019. The average EC was relative high in 2014–2015 and significantly higher than in 2019 (*P* = 0.0011).

The highest average concentrations of TSS and ISS were recorded in 2014 (17.4 mg L^−1^ and 7.6 mg L^−1^, respectively), which in 2019 statistically significantly decreased to 4.3 mg L^−1^ (*P* = 0.0325) and 0.9 mg L^−1^ (*P* = 0.0255), respectively. The most pronounced and statistically significant decreases in nitrate to 0.050 mg L^−1^ (*P* = 0.0026), calcium to 68.1 mg L^−1^ (*P* = 0.0016) and chlorine to 10.5 mg L^−1^ (*P* = 0.0003) in 2019 were also noted. The lowest average content of sodium in 2018 and 2019 (4.70 mg L^−1^ and 4.76 mg L^−1^, respectively) differed significantly in 2015 (69.83 mg L^−1^; *P* = 0.0009). Additionally, the highest concentrations of phosphates (0.024 mg L^−1^), TP (0.126 mg L^−1^) and NH_4_^+^ (0.116 mg L^−1^) were recorded in the first year of observation in the Kuźnica pond, and they were statistically lower in other years (*P* = 0.0071*, P* = 0.0135 and *P* = 0.0121, respectively).

In 2014–2019, the dominant components of trace elements in the Kuźnica pond were Si, Fe and Mn (Supplementary Table S3). Their highest concentrations were as follows: 16.78 mg L^−1^ (in 2016), 3.84 mg L^−1^ (in 2014) and 0.36 mg L^−1^ (in 2017), respectively. Statistically significant differences were noted only for Fe (*P* = 0.0028). The concentrations of other trace elements were characterized by moderate variability (Table 1). Among them, the highest ranges of concentration were for Al (1.14–2.97 µg L^−1^), Pb (0.42–1.06 µg L^−1^), Cu (1.52–2.42 µg L^−1^), Ni (2.01–3.01 µg L^−1^) and Zn (20.01–33.96 µg L^−1^). The average concentrations of Ag, As, Cd and Hg, were about 0.01 µg L^−1^ in most samples, while for Se it did not exceed this value. Thus, their content corresponded well to the best water quality class. Summing up, no toxic risk from the elevated content of trace elements was also noted.

**Table 1:** Content of trace elements in the Kuźnica clarification pond in 2014–2019 compared to threshold values and classification of water quality (Regulation of the Minister of Maritime Economy and Inland Navigation, 2019^a^).

Parameter	Range	Mean	± SD	Threshold value^a^	Water quality class^a^
Ag (µg L^−1^)	0.01–0.02	0.01	< 0.01	0.005 mg L^−1^	I–II
Al (µg L^−1^)	1.14–2.97	2.16	0.47	0.400 mg L^−1^	I–II
As (µg L^−1^)	0.01–0.03	0.20	< 0.01	0.050 mg L^−1^	I–II
Cd (µg L^−1^)	0.01–0.02	0.01	< 0.01	0.080 µg L^−1^	I–II
Cu (µg L^−1^)	1.52–2.42	1.94	0.27	0.050 mg L^−1^	I–II
Ni (µg L^−1^)	2.01–3.01	2.55	0.34	4.000 µg L^−1^	I–II
Pb (µg L^−1^)	0.42–1.06	0.73	0.19	1.200 µg L^−1^	I–II
Se (µg L^−1^)	< 0.01	< 0.01	< 0.01	0.020 mg L^−1^	I–II
Zn (µg L^−1^)	20.01–33.96	27.64	4.96	1.000 mg L^−1^	I–II
Hg (µg L^−1^)	0.01–0.02	0.01	< 0.01	–	–
Fe* (mg L^−1^)	0.32–5.80	1.78	1.26	–	–
Mn (mg L^−1^)	0.05–0.51	0.25	0.11	–	–
Si (mg L^−1^)	12.48–17.98	15.04	1.38	–	–

– not defined.

*Statistically significant differences at the significance level *P* = 0.0028 (see Supplementary Table S3 for detailed data).

**Figure 1:**
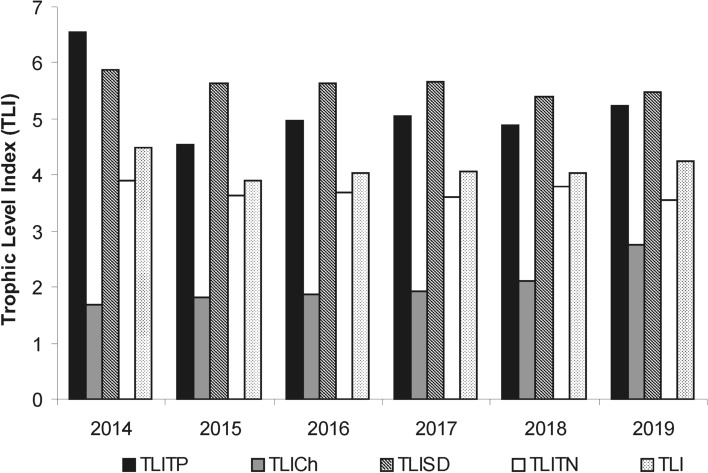
Trophic Level Index (TLI) and its partial indices based on TP (TLITP), TN (TLITN), SDD (TLISDD) and Chl *a* (TLICh) (averages from the growth season); trophic classification: 0–1, 1–2, 2–3, 3–4, 4–5, 5–6, 6–7 for ultra-microtrophic, microtrophic, oligotrophic, mesotrophic, eutrophic, supertrophic and hypertrophic, respectively.

The partial indices of TLI were markedly differentiated (Fig. 1), with the highest values of 4.9–6.5 and 5.4–6.0, respectively for TLI_TP_ and TLI_SDD_. They covered the classes from eutrophic to hypertrophic levels. The TLI_TN_ values varied (3.6–3.9) within the class characteristic for mesotrophic waters. The lowest values (1.7–2.5) and typical of microtrophic and oligotrophic levels were recorded for TLI_Chl_. Thus, the average values of partial indices pointed to levels from supertrophic to oligotrophic and confirming the general relationships as follows: TLI_SDD_ > TLI_TP_ > TLI_TN_ > TLI_Chl_. However, the average value of TLI for the whole study period (4.1) indicated a slightly eutrophic level in the Kuźnica clarification pond.

### Phytoplankton features and phytoplankton-based assessment

The phytoplankton assemblages consisted of seven phyla: Cyanobacteria, Bacillariophyta, Chlorophyta, Cryptophyta, Euglenozoa, Miozoa and Ochrophyta. In total, 79 phytoplankton species were found with gradually increase from year-to-year, i.e. from 10 species in 2014 to 46 species in 2019. The values of the Shannon Index ranged from 1.381 to 2.248, whereas evenness ranged from 0.436 to 0.594, except for 2014 (0.817).

**Figure 2:**
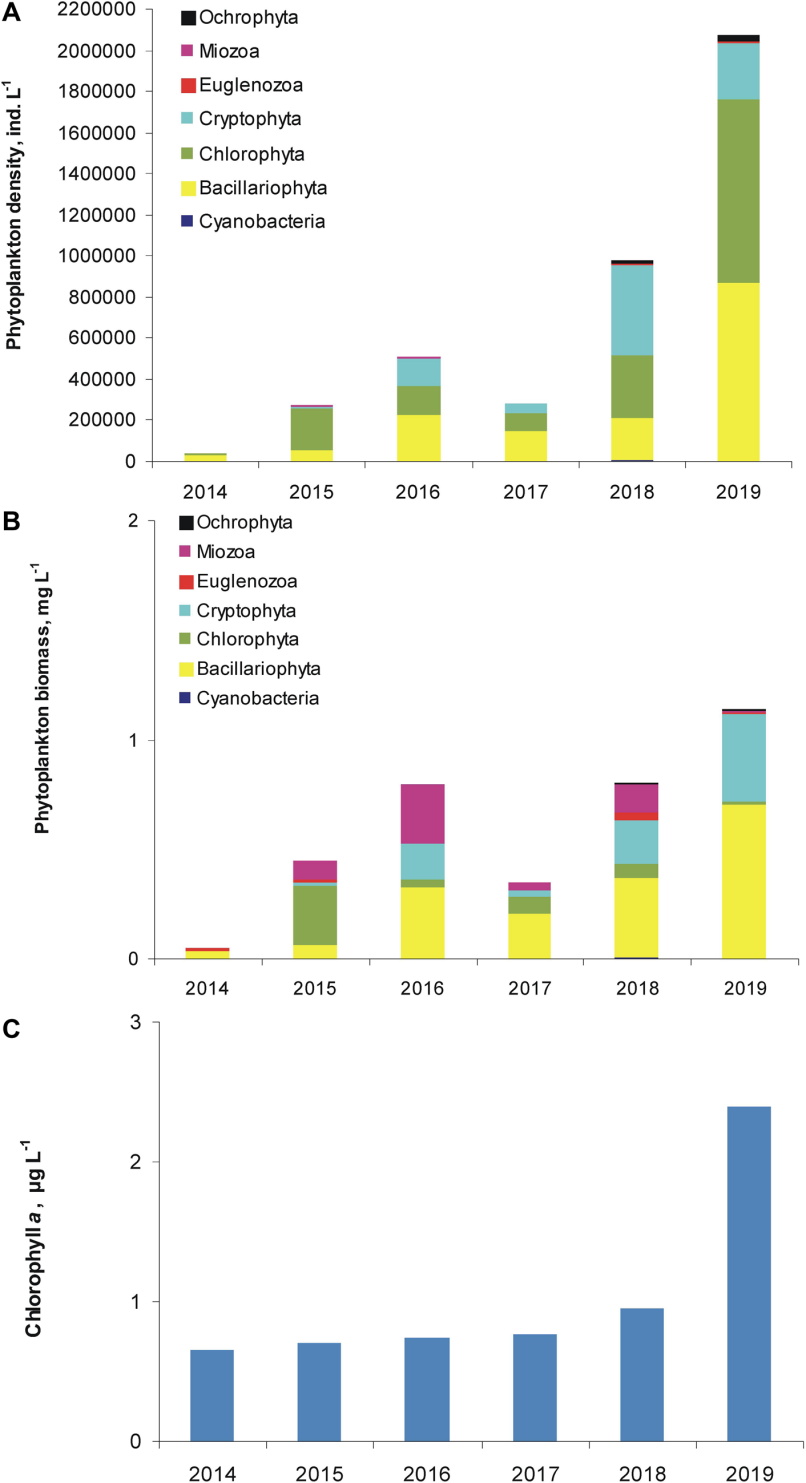
Phytoplankton density (**A**), biomass (**B**), and chlorophyll *a* content (**C**) in the Kuźnica clarification pond in 2014–2019 (seasonal averages).

The total phytoplankton density gradually increased from 0.40 × 10^5^ ind. L^−1^ in 2014 to 20.78 × 10^5^ ind. L^−1^ in 2019, on average (Fig. 2A). Diatoms (19–76% of the total density), chlorophytes (14–74%) and cryptophytes (3–44%) dominated the phytoplankton. The total biomass ranged then from 0.05 to 1.39 mg L^−1^, with seasonal averages of 0.05–1.14 mg L^−1^ (Fig. 2B). Diatoms, representing 41–67% of the total biomass dominated the phytoplankton assemblages throughout the almost whole study period. The exception was in 2015 when small chlorophytes formed the highest biomass (61% of the total biomass), and dinoflagellates and diatoms about 19% and 14%, respectively. Besides diatom dominance, euglenoids and cryptophytes had a share of 21% and 7%, respectively in 2014. In 2016–2018, in turn, dinoflagellates had 10–34%, cryptophytes 8–25% and chlorophytes 5–24%, and in 2019 only cryptophytes (with 35%) co-dominated with diatoms. Simultaneously with gradually increase in phytoplankton density and biomass, the chlorophyll *a* content also increased from 0.7 to 2.4 µg L^−1^, on average in a temporal scale (Fig. 2C). However, during the growth season it changed in a generally narrow range, from 0.4 to 4.5 µg L^−1^.

The most numerous were the small-sized species, i.e. diatoms: *Cyclotella meneghiniana* Kützing, *Nitzschia palea* (Kützing) W.Smith, green algae: *Chlamydomonas reinhardtii* P.A.Dangeard, *Monoraphidium contortum* (Thuret) Komárková-Legnerová, *Kirchneriella irregularis* (G.M.Smith) Korshikov, and cryptophytes: *Plagioselmis nannoplanctica* (Skuja) G.Novarino, I.A.N.Lucas & Morrall, *Cryptomonas erosa* Ehrenberg (Table 2A). They represent the coda of different habitat-related functional groups: C, D, X2, X1, F and Y. The dominance structure based on biomass was a slightly different. However, the highest biomass formed also the above mentioned species of the genera *Chlamydomonas* and *Cyclotella* (Table 2B). A relatively high biomass formed the large-sized dinoflagellates: *Ceratium hirundinella* (O.F.Müller) Dujardin and *Peridinium cinctum* (O.F.Müller) Ehrenberg which are the representatives of codon L_O_, and diatom *Ulnaria ulna* (Nitzsch) Compère of codon MP. Besides this, a high share in 2014 had euglenoid *Trachelomonas armata* (Ehrenberg) F.Stein (codon W2) and diatom *Staurosira construens* Ehrenberg (codon MP). Exceptionally in 2019, the representative of codon U chrysophytes *Uroglenopsis americana* (G.N.Calkins) Lemmermann formed relatively high density and biomass.

**Table 2:** The dominant species (mean % for the growth season) in phytoplankton density (A) and biomass (B) in the Kuźnica clarification pond in 2014–2019.

Species	Phylum	FGs	2014	2015	2016	2017	2018	2019
**(A) Relative density**								
*Cyclotella meneghiniana*	Bacillariophyta	C	35	13	39	49	15	65
*Nitzschia palea*	Bacillariophyta	D	23	6	3	1	2	2
*Chlamydomonas reinhardtii*	Chlorophyta	X2	–	56	–	13	14	4
*Kirchneriella irregularis*	Chlorophyta	F	–	19	26	16	11	1
*Monoraphidium contortum*	Chlorophyta	X1	10	–	–	2	1	37
*Cryptomonas erosa*	Cryptophyta	Y	3	1	9	1	3	1
*Plagioselmis nannoplanctica*	Cryptophyta	X2	–	3	17	14	41	16
*Uroglenopsis americana*	Ochrophyta	U	–	–	–	–	–	8
**(B) Relative biomass**								
*Cyclotella meneghiniana*	Bacillariophyta	C	39	10	34	54	9	40
*Staurosira construens*	Bacillariophyta	MP	18	–	–	–	1	–
*Ulnaria ulna*	Bacillariophyta	MP	–	1	1	1	25	3
*Nitzschia palea*	Bacillariophyta	D	10	2	1	1	1	2
*Chlamydomonas reinhardtii*	Chlorophyta	X2	–	60	–	18	2	1
*Cryptomonas erosa*	Cryptophyta	Y	7	2	16	1	15	14
*Plagioselmis nannoplanctica*	Cryptophyta	X2	–	1	4	4	7	6
*Trachelomonas armata*	Euglenozoa	W2	21	–	–	–	–	–
*Ceratium hirundinella*	Miozoa	L_O_	–	–	27	-	14	–
*Peridinium cinctum*	Miozoa	L_O_	–	19	6	9	1	–
*Uroglenopsis americana*	Ochrophyta	U	–	–	–	–	–	11

*FGs* functional groups (Reynolds et al. 2002, Padisák et al. 2009) and habitat template of coda, *C* eutrophic small- and medium sized lakes with species sensitive to the onset of stratification, *D* shallow turbid waters including rivers, *F* clear, deeply mixed meso-eutrophic lakes, *X1* shallow, *eu* hypertrophic environments, *X2* shallow, meso-eutrophic environments, *Y* this codon, mostly including large cryptomonads but also small dinoflagellates, refers to a wide range of habitats, which reflect the ability of its representative species to live in almost all lentic ecosystems when grazing pressure is low, *MP* frequently stirred up, inorganically turbid shallow lakes, *W2* meso-eutrophic ponds, even temporary, shallow lakes, *L*_*O*_ deep and shallow, oligo to eutrophic, medium to large lakes, *U* stratifying oligotrophic and mesotrophic lakes, where nutrient resources are exhausted in the upper layers but still available in the darker deep ones, the essential adaptation under these conditions is the combination of motility with large size, – not observed.

The ecological conditions in the clarification pond were estimated according to the phytoplankton-based assessment of ecological potential. The values of partial metrics (MTB, MCB, MC) were very stable and amounted close to 1.00 (Supplementary Table S4). The final multimetric PMPL indicated, thus, a maximum ecological potential throughout the whole study period. It was consistent to relative small phytoplankton biomass and chlorophyll *a* content, and their insignificant fluctuations did not affect any changes in the ecological classification.

### Relationships between phytoplankton and environmental variables

**Figure 3:**
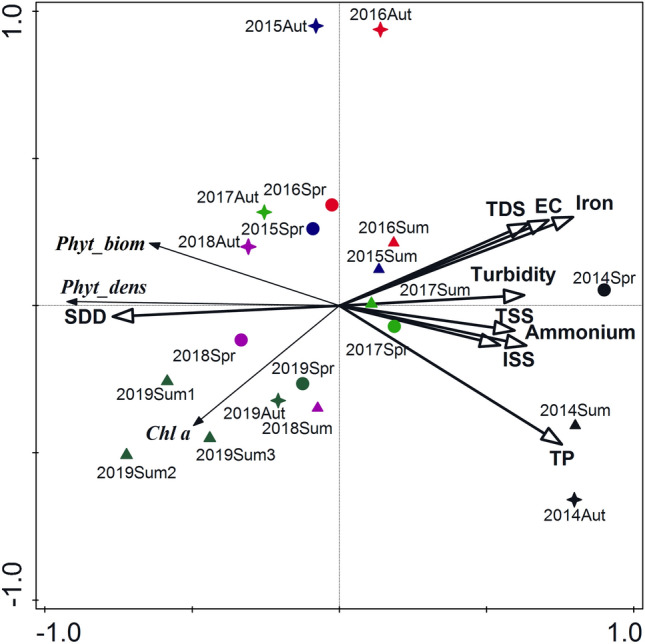
Triplot diagram of RDA for phytoplankton density, biomass, and chlorophyll *a* content and main environmental variables in the Kuźnica clarification pond in 2014–2019. *Phyt_biom* phytoplankton biomass, *Phyt_dens* phytoplankton density, *Chl a* chlorophyll *a* content, *EC*electrical conductivity, *TDS* total dissolved solids, *TSS* total suspended solids, *ISS* inorganic suspended solids, turbidity, *SDD* Secchi disk depth, ammonium, *TP* total phosphorous, *Fe* iron.

The relationships between phytoplankton and environmental variables were determined in RDA ordination with a total variation of 38.18 and explanatory variables accounting for 81.9% of the variance. The sum of all the canonical eigenvalues was 0.8195. The first two components of RDA explained 81.6% of the total variance of response data including the first axis accounted for 80.3%. The phytoplankton density and biomass were negatively correlated with total phosphorus, ammonium, TSS and ISS (Fig. 3). The negative relationships were also found between chlorophyll *a* content and iron, EC and TDS. The positive relationships, in turn, were only between phytoplankton features and SDD. The RDA ordination confirmed also a relatively high similarity of both phytoplankton and environmental variables primarily in 2018 and 2019 and selected samples from 2015 to 2017. The indicators coming from the all seasons in 2014 and autumn in 2015–2016 were completely separated from the other samples.

### New ecological opportunities for charophytes

The overall phytosociological observations were conducted from 2014 up to date, confirmed that the single-species community of charophyte *Nitella mucronata* (Supplementary Fig. S1) was identified for the first time in an early spring of 2019. After that, it began to grow rapidly to the end of the growth season. In spring, the biomass of *N. mucronata* was slightly diversified between different sites and it ranged from 26.58 to 32.60 g DW m^−2^ in the inflow and outflow sub-zones, respectively (Table 3). A seasonal increase of the average biomass was found in each zone of the pond. The more pronounced increase was observed in the outflow zone. There was recorded a significant increase of *N. mucronata* biomass (*P* < 0.01), from 32.60 to 323.39 g DW m^−2^ in spring and autumn, respectively. Only, in the inflow sub-zone the increase of the biomass (26.58 g DW m^−2^, 43.71 g DW m^−2^ and 152.35 g DW m^−2^ in spring, summer and autumn, respectively) was statistically insignificant. Generally, a statistically significant increase of the average biomass of the charophyte from 29.01 to 246.95 g DW m^−2^ (*P* < 0.001) was recorded throughout the whole growth season.

**Table 3:** Seasonal variability of *Nitella mucronata* biomass in sub-zones of the main water zone of the Kuźnica clarification pond in 2019.

Season	Mean ± SD (g DW^a^ m^−2^)			
	Inflow^b^	Central^c^	Outflow^d^	Total area^e^
Spring	26.58 ± 1.05^A^	27.83 ± 1.56^A^	32.60 ± 1.23^A^	29.01 ± 2.94^A^
Summer	43.71 ± 24.02^A^	77.24 ± 6.62^A^	93.71 ± 6.74^B^	71.56 ± 25.58^B^
Autumn	152.35 ± 56.62^A^	265.10 ± 28.21^B^	323.39 ± 33.54^C^	246.95 ± 82.87^C^

Values with the different superscripts are significantly different among seasons confirmed by non-parametric Kruskal–Wallis test (*p* < 0.05).

^**a**^Dry weight; ^b^*H* = 2.05, *N* = 15, *df* 2, *p* > 0.05; ^c^*H* = 7.13, *N* = 15, *df* 2, *p* < 0.05; ^c^*H* = 22.13, *N* = 15, *df* 2, *p* < 0.01; ^e^*H* = 79.78, *N* = 15, *df* 2, *p* < 0.001.

### Sequential ecosystem changes

**Figure 4:**
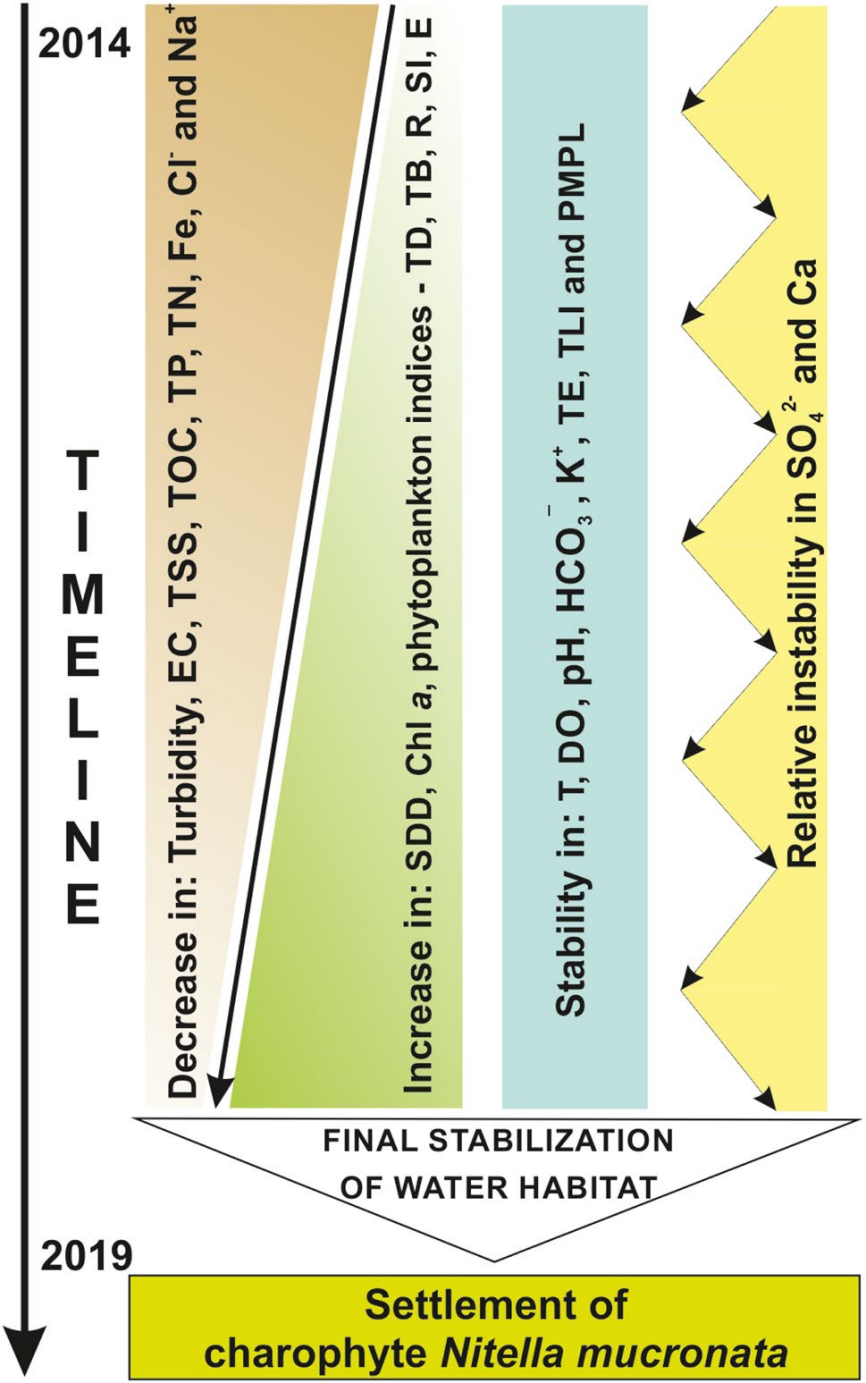
Sequential ecosystem changes and settlement potential in clarification pond. *EC* electrical conductivity, *TSS* total suspended solids, *TOC* total organic carbon, *TP and TN* total phosphorus and nitrogen, *Fe* total iron, *SDD* Secchi disk depth, *Chl a* chlorophyll *a* content, *TD and TB* total density and biomass, *R* richness, *SI* Shannon index, *E* evenness, *T* temperature, *DO* dissolved oxygen, *TE* trace elements, *TLI* trophic level index, *PMPL* Phytoplankton Metric for Polish Lakes.

Studying the 6-year-long sequential ecosystem changes and settlement potential, it can be summarized as follows (Fig. 4):

1. gradual decrease in turbidity, electrical conductivity, suspended solids, carbon, phosphorus, nitrogen, iron, and some ions;
2. gradual increase in chlorophyll *a*, phytoplankton indicators (such as: density, biomass, richness and biodiversity) and water transparency;
3. stability in temperature, oxygen, pH, bicarbonate, potassium, most of the trace elements, and trophic level and ecological potential;
4. relative instability in sulfates and calcium;
5. final stabilization of water habitat and a new ecological opportunity for the settlement of charophyte *Nitella mucronata*.

The processes that have accompanied the ecosystem changes included primarily the effective sedimentation, nutrient-iron bounding and their release to the bottom, decreased bioavailability for primary producers. In consequence, an effective limitation of the phytoplankton growth can successfully maintain the maximum ecological potential on the long-term scale.

## Discussion

The Kuźnica clarification pond gives the opportunity to study a unique water body concerning the possible biotic live. This pond was selected for the studies on the possibility to colonize by primary producers, then, other organisms with an appearance of new species, and consequently the changes in an ecological potential against the variability of physical and chemical parameters of the mine water. The relatively low TDS content (not exceeding 529 mg L^−1^) and slightly increased conductivity, which should be associated with the increased content of sulphates, carbonates and chlorides, proved the origin of the freshwater supplied to the clarification pond. This state reflects “specific” geochemical conditions, and a high concentration of calcium and carbonates stabilized the slightly alkaline pH of the water in the Kuźnica pond. According to Gogacz^15^, freshwater of the mesozoic zone are drained through the Szczerców open pit drainage system. The mineralization does not increase with the depth of the open pit drained levels, and a bicarbonate-calcium character is typical, with only periodic increases in the concentration of the chloride-sodium complex and pH.

The general trend of changes in hydrochemical variables was characterized by: (1) the highest concentrations of total phosphorus and ammonium nitrogen in the first year of study and then their stabilization; (2) a gradual annual decrease in the average concentrations of most studied parameters in the second half of the study period (i.e. turbidity, PO_4_^3−^, Na^+^), and especially in 2019 (i.e. EC, TSS, ISS, NO_3_^−^, Ca^2+^, Cl^−^, Fe_Tot_), and (3) gradual increase in water transparency (SDD) with the highest values in 2018–2019. The above mentioned changes could be directly related to the progressive exploitation of lignite deposits. As a result, the exposure to transitional rocks within the Mesozoic—neogen contact zone has then increased. According to Pękala^16^, these rocks consist mainly of silica and calcium oxide as well as calcite with variable iron and manganese content. In good oxygen conditions, iron and manganese significantly influenced the turbidity and water transparency in the Kuźnica pond. Suspended solids, which are characteristic of the aquatic environment of the clarification pond, were, thus, the transporter for the precipitate of iron and manganese hydroxides. Similar results were described by other authors^17,18^.

The variability of geochemical phenomena in the Bełchatów mine was favored by the local tectonics of the mesozoic substrate, and the presence of halite diapir located between two exploited openings^16,19^. The research also showed that sodium chloride anomalies (effect of water ascension) and manganese-iron anomalies were noted in some wells of the drainage system. The hydrochemistry of the Kuźnica clarification pond and the water quality in the surface drainage system were also influenced by other factors related to the progress of the exploitation front. Then, the new layers of sediments were exposed and their structure was loosened. The progresses of the operating front and controlled shutting down of the drainage wells resulted in the groundwater restoration^19^. Additionally, the reconstruction of the groundwater level is constantly renewed around the Szczerców opencast mine. This process was initiated in 2012, i.e. before the construction of the Kuźnica pond. At that time, the formation of an internal heap began, and the rebuilding rate of the groundwater level depended on its precipitation infiltration. Thus, the observed changes and stabilization of the chemical composition of water in the Kuźnica pond were directly influenced by many interrelated factors.

In Poland and especially in the area of studied water body, there was not recorded any elevated content of heavy metals. In current studies, the relatively low content of studied trace elements allows the Kuźnica clarification pond to be classified as waters having at least the second quality class, i.e. good ecological potential in accordance with the Polish Regulation^20^. There were no cases of exceeding the permissible concentration limits of trace elements. Generally, the mining activities have become the historically and globally important contributors to heavy metal pollution of many water bodies. In Poland, and especially in the area of mining activities, such situation with a high content of heavy metals was not recorded yet^16,21,22^.

The newly-formed clarification pond studied here was generally characterized by very low phytoplankton growth and diversity similarly to the findings recorded in the other newly-created post-mining lakes in Poland^22^. However, the 6-year-long ecosystem changes were related to a very slow but gradual increase in chlorophyll *a* content and phytoplankton features, i.e. density, biomass, richness and biodiversity. Current study, and just like previous study on the possibility of using this pond to extensive aquaculture^13^, suggest that phytoplankton could be limited by a large amount of suspended solids during both the mechanical and shading processes. These include primarily the sinking of phytodetrital aggregates and a light limitation^23,24^, as well as negative relations with zooplankton. The negative relationship between phytoplankton features (biomass, density, chlorophyll *a*) and environmental variables (total phosphorus, ammonium, TSS, ISS, TDS, iron and EC) was also confirmed. Simultaneously, the positively-related to phytoplankton water transparency tended to gradually increase, and it was related rather to the decrease of suspended solids content but not to phytoplankton growth. A phenomenon of reduced visibility in the water column by organic matter was also characteristic of the post-mining lakes^25^.

Low values of total biomass, cyanobacterial biomass and content of chlorophyll *a* in the whole study period of 2014–2019 were typical of high water quality, i.e. maximum ecological potential of the Kuźnica clarification pond in accordance with the ecological classification of European water bodies^7^, and close to the unimpaired state, i.e. reference conditions common for European lakes^26^. The biodiversity Shannon index pointed at moderately or slightly polluted waters based on classifications given in Napiórkowska-Krzebietke^27^, whereas the final TLI indicate a slightly eutrophic level. Based on functional classification^28,29^, the dominant species of are associated with different habitats. The most abundant diatoms are characteristic for eutrophic, shallow, turbid (primarily inorganically) water bodies, next, the small-sized green algae are typical for shallow, meso-eu-hypertrophic environments or even clear, deeply mixed meso-eutrophic lakes similarly to cryptomonads which refer to a wide range of habitats. The large-sized dinoflagellates, i.e. *Ceratium hirundinella* and *Peridinium cinctum* prefer deep and shallow, from oligo to eutrophic ones. In opposite, the euglenoid *Trachelomonas armata* occurs primarily in meso-eutrophic small ponds, and the euglenophytes often exist in water containing organic pollution^30^.

Furthermore, in 2019 i.e. in the 6th year of phytosociological observations, the clarification pond was inhabited by *N. mucronata*. This charophyte is included as one of the rarest Polish charophytes and considered as critically endangered or extinct species on the Red list of algae^31–33^. Currently, *N. mucronata* is also found in shallow artificial water bodies, which are characterized by trophic poverty and good light conditions^33,34^. For the growth of charophytes, an increased concentration of calcium ions and a slightly alkaline water pH are very important^35,36^, and such conditions are met in the water of the Kuźnica pond. However, the occurrence of *N. mucronata* in the turbid environment has not yet been recorded. The most charophytes cannot grow in water that is permanently very turbid and enriched in nutrients, but some of them can photosynthesize at low light intensities, or can survive in some periods of turbid water^37^. Therefore, the abundant occurrence of *N. mucronata*'s monospecies community in the Kuźnica clarification pond proves the high colonization potential for this species. Its maximum biomass recorded in autumn 2019 (nearly 247 g DW m^−2^) was relatively high and similar to the results from the other study^38^. This can prove the wide tolerance of *N. mucronata* for hydrochemical parameters and light availability. It is worth noting that Kuźnica pond was poor in nutrients, and enhanced water turbidity was not a result from the increased chlorophyll *a* content or phytoplankton biomass but iron hydroxides had a significant influence on water transparency and turbidity. The findings of Fontanini et al.^39^ showed that *N. mucronata* is perfectly suited for environments with increased iron content. The research showed that not only the light availability, but also the factors which determine the penetration of light in the water can affect the expansion of the charophyte. Thus, the appearance of *N. mucronata* was not only the result of the improvement of light conditions but also of the favourable water chemistry. Similarly to the findings of Blindow et al.^9^, the rapid development of *N. mucronata* in the clarification pond was the result of the stabilization of environmental variables and the ecological potential. Such stabilization in the shallow Kuźnica pond was a result of separating the sediments from the water which highly limited their resuspension. The reducing of resuspension has been a key contribution of macrophytes to an improvement of the water transparency in shallow water bodies^40^. The ability to act as a nutrient sink is also a stabilization factor which can cause the decrease in the nutrient availability for phytoplankton and epiphyton^41^.

The 6-year-long sequential ecosystem changes and settlement potential confirmed that natural processes allowed the turbid water of the shallow pond to reach the phase of stabilized environmental conditions. The key role of *Nitella mucronata* in stabilizing the maximum ecological potential in a newly-formed clarification pond was also suggested. Such waters were characterized by environmental variables typical of natural lakes. In previous studies, a limited phytoplankton and zooplankton growth with good growth rate of omnivorous *L. idus* were confirmed^13^. Now, in this study, we indicate that environmental conditions of clarification pond can support also the proper functioning of ecosystem. The multi-seasonal results proved also that the pond fed with water from the opencast mine drainage system can be safely used for utility purposes, one of which is recreational fishing. All changes in trophic state or ecological status have an impact on the habitat conditions and the growth rate of many fish species, including those in artificial lakes^42,43^. Therefore, the stabilization of low trophic level and maximum ecological potential is an inspiration for further research on fish habitat in such ponds.

## Conclusions

The 6-year-long research on ecosystem changes in the Kuźnica clarification pond confirmed a final stabilization in low nutrient enrichment and non-algal turbidity-related variables. Generally, the limited phytoplankton growth and especially low cyanobacteria biomass and chlorophyll *a* content but with a gradual and slight increase were recorded throughout the whole study period. The phytoplankton assemblages were dominated by species with different habitat requirements including meso-eu-hypertrophic turbid waters as well as oligotrophic ones. Diatoms of the genera *Cyclotella, Nitzschia*, *Ulnaria* and *Staurosira*, green algae of the genera *Chlamydomonas*, *Monoraphidium* and *Kirchneriella*, and cryptomonads of the genera *Plagioselmis* and *Cryptomonas* were dominants. The final phytoplankton-based assessment indicated a maximum ecological potential whereas TLI-based assessment indicated a slightly eutrophic level in the Kuźnica clarification pond. A low content of trace elements and low biomass of toxic or potentially toxic cyanobacteria demonstrated a very low toxic risk.

Our study confirmed the possibility to inhabit and abundantly grow of the sensitive charophyte considered as critically endangered or extinct species *Nitella mucronata* in water of clarification pond. *N. mucronata* played an important role in the stabilizing the environmental conditions and maintaining at least good ecological potential in a newly and formed clarification pond. Therefore, the clarification pond can support populations of charophyte species, and provide a new habitat for aquatic species that is less available in naturally occurring shallow and turbid water bodies of the region.

## Materials and methods

### Study area

**Figure 5:**
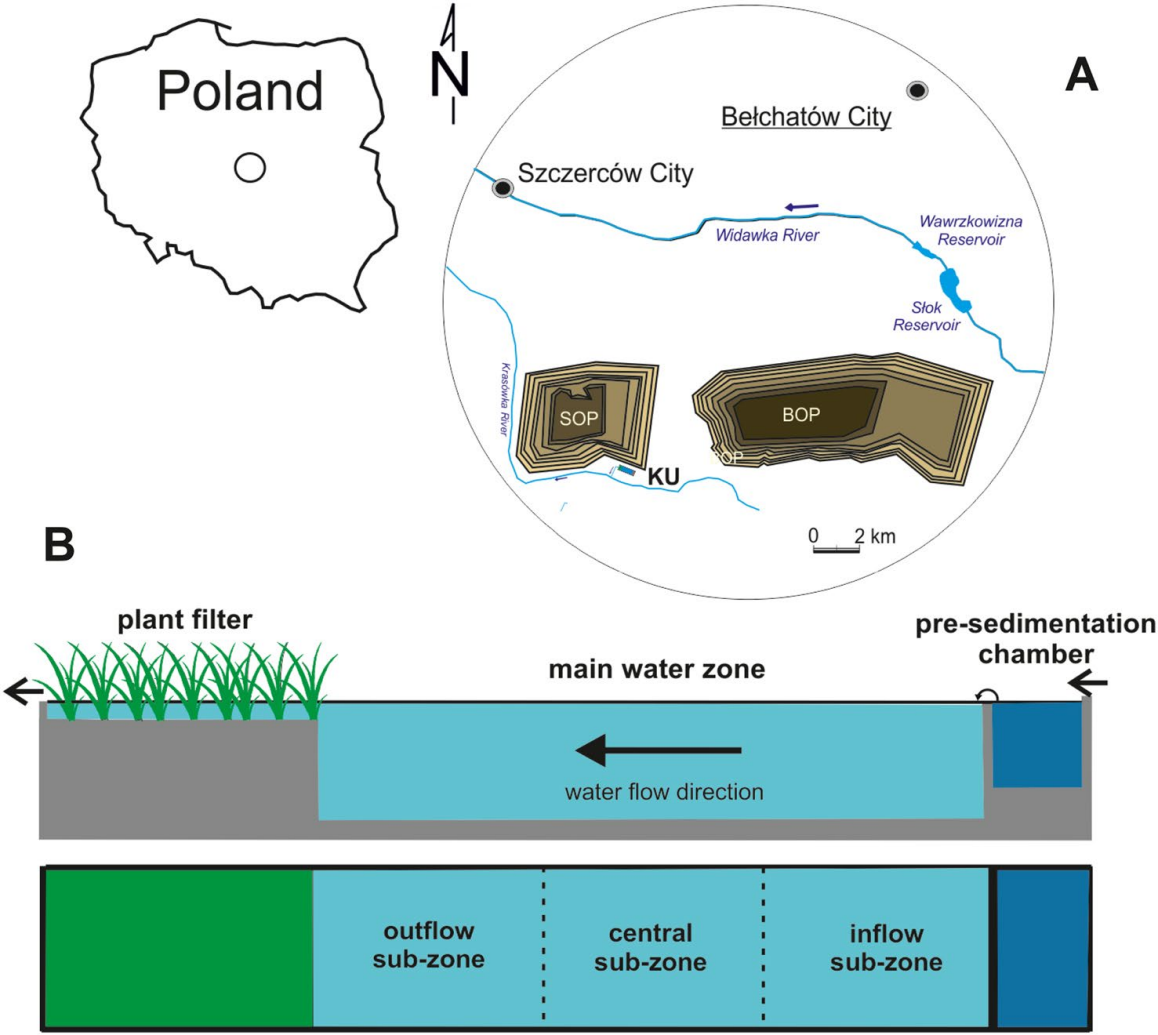
Research site location (**A**) and scheme of the Kuźnica clarification pond in a cross-section (**B**). *KU* Kuźnica clarification pond, *SOP* Szczerców open pit, *BOP* Bełchatów open pit.

The study site is a newly-formed pond created in 2013 to pre-treat water from dewatering system of the largest brown coal strip mine in Poland so-called “Bełchatów” (51° 13′ 29.5″ N, 19° 9′ 26.5″ E, Fig. 5A). Its basic role is a technical function in reducing the excess of suspended matter throughout the sedimentation process, thus, it can be called as clarification pond. The Kuźnica clarification pond receives the waters originating from different depths of the surface drainage system (surface and ground waters from shallow wells mixed in variable proportions) of the open pit in Szczerców. The pond is surrounded by stone embankments equipped with 5 m wide crests and sloping edges of 1:2. The surface area of 2.60 ha is divided into three technological parts, i.e. pre-sedimentation chamber (area of 0.10 ha), main water zone (area of 1.75 ha with maximum depth of 2.8 m) and plant filter (area of 0.75 ha with mean depth of 0.5 m) (Fig. 5B). The third zone includes emergent macrophytes, primarily *Typha latifolia* L., *Phragmites australis* (Cav.) Trin. ex Steud., *Glyceria maxima* (Hartm.) Holmb., *Carex acutiformis* L., *Acorus calamus* L. and *Phalaris arundinacea* L. The water outflow (1.5 m^3^ s^−1^; retention time of 10 h, on average) into discharge canal is located at the end of this zone.

### Physical and chemical parameters and analyses

The study comprises the period of 2014–2019. The rationale for samplings was to obtain a representative site, which concerned the central part of main water zone in the clarification pond. The water samples for physical and chemical parameters were collected at 1 m depth (below the surface layer) including three-four times during the whole growth season. Field measurements were done in situ in parallel to sampling occasions. Water temperature (T), dissolved oxygen (DO) content and oxygen saturation (%) were measured using the Multi-Parameter Water Quality Sonde YSI 6600 V2. The pH, electrical conductivity (EC) and total dissolved solids (TDS) were measured using the digital multimeter HQ30D. Water transparency was using Secchi disk and expressed as Secchi disk depth (SDD).

The laboratory analyses of physical and chemical parameters concerned: total suspended solids (TSS), inorganic suspended solids (ISS), biochemical oxygen demand (BOD), total organic carbon (TOC), chlorophyll *a* (Chl *a*), total nitrogen (TN), total phosphorous (TP), silicon (Si), calcium (Ca), iron (Fe_Tot_), manganese (Mn), phosphate (PO_4_^3−^), nitrate (NO_3_^−^), ammonium (NH_4_^+^), bicarbonate (HCO_3_^−^), sulfate (SO_4_^2−^), and other selected ions (Mg^2+^, Cl^−^, Na^+^, K^+^). All these analyses were conducted according to standard methods^44^. Furthermore, the content of selected trace elements, primarily: Fe, Mn, Si, Ag, Al, As, Cd, Cu, Hg, Ni, Pb, Se, Si, Zn was determined with the accuracy of 0.01 μg L^−1^ according to the methodology based on an elementary coupled plasma ionization mass spectrometry (ICP-MS) using an emission spectrometer Elan ICP-MS (Perkin Elmer). The trace elements’ selection for the determinations was made based on the results of the hydrogeochemical background performed for the area covering the mining activities^16,21^. Such a range of hydro-analyses coincides with the cyclic monitoring of the mine water quality supervised by the Polish National Hydrogeological Service^15^.

### Biological parameters and analyses

The integrated phytoplankton samples at 1 m depth were also taken out in the three-five times regime throughout the growth season, i.e. in spring, summer and autumn. The samples for taxonomic analysis were additionally taken using the plankton net with 10 μm mesh size. The quantitative analysis covering phytoplankton density and biomass was performed using an inverted microscope according to standard Utermöhl method^45^. The counting organisms (single cells, colonies or filaments) were examined at different magnifications: 100×, 200× and 400× for large-, medium-, and small-sized taxa, respectively. The qualitative analysis (taxonomic identification) was performed using a light microscope at magnifications of 200×, 400×, and 1000× with oil immersion. The total biomass and biomass of phytoplankton taxa were calculated on the basis of the cell biovolume measurements according to standard and revised method^46^. Taxonomic identifications were based on the latest references and verified according to currently accepted taxonomic names given in AlgaeBase^47^.

Phytosociological observations were also conducted from 2014 to 2019. In 2014–2018, no submerged macrophytes were found at the bottom of the main water zone. At the end of February 2019, charophyte *Nitella mucronata* (A.Braun) Miquel was observed for the first time and the expansion of this single-species *Nitelletum mucronatae* community was, thus, monitored. Taxonomic identification was made according to Pełechaty and Pukacz^48^. The observations were carried out on a total of 15 sites in the main water zone, including 5 sites each in the sub-zones: inflow (within the vicinity of the pre-sedimentation chamber), central and outflow (within the vicinity of the plant filter). Charophyte was collected regularly at a monthly interval throughout the growth season, i.e. spring, summer and autumn, using a bottom sampler according to Ekman-Birge with grasping area of 0.04 m^−2^. Samples were cleaned of sediments, identified and dried in an air-forced circulation dryer at 70 °C until a constant weight was obtained. The biomass of submerged macrophyte was expressed in grams of dry weight (DW) per 1 m^−2^ of bottom area.

### Functional, ecological and trophic classifications

The species richness expressed as the total number of phytoplankton species, and diversity indices, i.e. Shannon Index^49^ and evenness^50^ were chosen to describe phytoplankton biodiversity. The values of diversity indices were calculated based on species density. Determination of the main phytoplankton representatives of functional groups was based on the functional classification^28,29^.

Due to a lack of concern such a water body in Polish regulations but also across the European countries^51^, in the ecological potential assessment of the Kuźnica clarification pond the criteria were assumed as follows:

- small, shallow, non-stratified, artificial water body,
- catchment area has no impact, it could be the most similar to very low impact,
- (Schindler's ratio, SR—ratio of catchment area and lake volume, SR = 0 ≈ SR < 2),
- high Ca content > 25 mg dm^−3^,
- sampling regime: three-four times throughout the growth season,
- the equations (1–5) for assessment were adapted Napiórkowska-Krzebietke et al.^52^, and references therein.

The Trophic Level Index (TLI) according to Burns et al.^53^ was selected for trophic state determination. It comprises four partial indices based on total phosphorus (TP), total nitrogen (TN), Secchi disk depth (SDD) and chlorophyll *a* (Chl *a*).

### Statistical analyses

Non-parametric analysis of variance (Kruskal–Wallis test) was used to assess the differences between years concerning the physical and chemical parameters of the water and a biomass of submerged macrophyte in the clarification pond. All the analyses were performed with Statistica 13.0 for Windows, Statsoft, Tulsa. The level of significance was set to a *P* < 0.05.

The relationships between phytoplankton characteristics and environmental parameters were tested with the redundancy analysis (RDA) of canonical ordination. The Monte Carlo permutation test (999) was then used. All tested parameters were standardized using log (x + 1)-transformation. Response data were compositional and had a gradient of 0.1 SD units long, thus, a linear method was recommended. The explanatory variables were additionally chosen after the analysis of variance inflation factor (VIF) and variables that had a VIF smaller than 10. The RDA was performed for phytoplankton characteristics (biomass, density and chlorophyll *a*) and nine environmental variables: EC, TDS, TSS, ISS, turbidity, SDD, ammonium, TP and Fe.

### Ethical approval

The study on charophyte *Nitella mucronata*, which belongs to macroalgae, complies with local and national regulations. It was conducted outside the natural aquatic ecosystems and outside the habitats covered by forms of area protection. Samples were taken in artificial water body located in the area of active mining site. In cooperation with the PGE Mining and Conventional Power Generation SA Branch KWB Bełchatów, for collection of charophyte samples, all relevant permits and permissions have been obtained.

## Supplementary Information

Supplementary Information 1.
