# Supplementary material for: A new charophyte habitat with a stabilized good ecological potential of mine water
Source: Sci Rep. 2021 Jul 15;11:14564. doi: 10.1038/s41598-021-93827-z (PMC8282622; doi:10.1038/s41598-021-93827-z)
Supplement: Supplementary file 1 — Supplementary Information 1. [file 41598_2021_93827_MOESM1_ESM.docx]

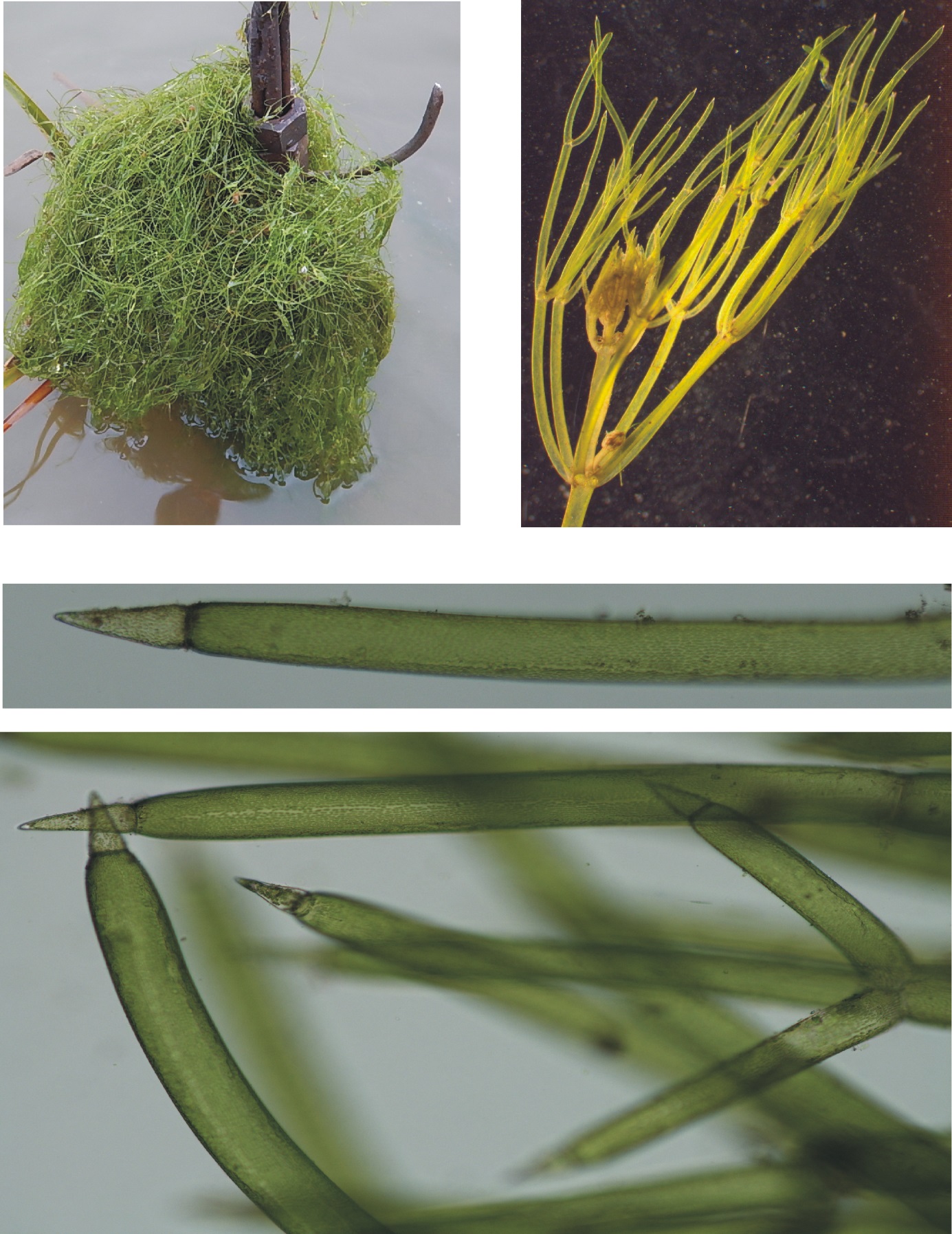


**Supplementary Fig. S1**. Macro- and microscopic (at magnification of 100×) view of *Nitella mucronata* inhabiting the Kuźnica clarification pond.

**Supplementary Table S1**. Seasonal variability of temperature and dissolved oxygen in water of the Kuźnica clarification pond in 2014-2019

| Season | T (°C) | | DO^1^ (mg L^-1^) | | DO^2^ (%) | |
| --- | --- | --- | --- | --- | --- | --- |
|  | Average | SD | Average | SD | Average | SD |
| Spring | 15.3 | ± 1.9 | 9.5 | ± 0.6 | 97.3 | ± 1.9 |
| Summer | 19.7 | ± 0.9 | 9.1 | ± 0.4 | 103.7 | ± 3.7 |
| Autumn | 15.8 | ± 2.5 | 9.5 | ± 0.9 | 98.2 | ± 2.1 |
| Winter | 11.1 | ± 1.3 | 10.3 | ± 1.1 | 96.3 | ± 2.4 |

T – temperature, ^1^ oxygen content, ^2^ oxygen saturation, SD - standard deviation

**Supplementary Table S2.** Physicochemical parameters of water in the Kuźnica clarification pond in 2014-2019. Values with the different superscripts are significantly different among the years by non-parametric Kruskal–Wallis test (*p* < 0.05)

| Parameters | 2014 | | 2015 | | 2016 | | 2017 | | 2018 | | 2019 | |
| --- | --- | --- | --- | --- | --- | --- | --- | --- | --- | --- | --- | --- |
|  | Average | Range | Average | Range | Average | Range | Average | Range | Average | Range | Average | Range |
| pH | 7.8 | 7.5-8.1 | 8.0 | 7.6-8.3 | 7.9 | 7.5-8.3 | 8.0 | 7.4-8.4 | 7.9 | 7.5-8.1 | 7.9 | 7.7-8.1 |
| **^1^** SDD (m) | 0.42**^A^** | 0.27-0.50 | 0.52**^AB^** | 0.33-0.65 | 0.55**^AB^** | 0.34-0.70 | 0.56**^AB^** | 0.37-0.75 | 0.69**^B^** | 0.55-0.85 | 0.68**^B^** | 0.60-0.75 |
| **^2^** Turbidity (NTU) | 40.7**^A^** | 16.0-78.0 | 38.1**^A^** | 15.0-65.0 | 37.3**^A^** | 21.0-54.0 | 11.8**^B^** | 6.0-17.0 | 12.3**^B^** | 6.0-27.0 | 14.0**^B^** | 10.0-21.0 |
| **^3^** EC (µS cm^-1^) | 771.8**^A^** | 745.0-786.0 | 771.0**^A^** | 702.0-884.0 | 712.2**^AB^** | 541.0-918.0 | 644.8**^AB^** | 530.0-736.0 | 574.5**^AB^** | 478-701 | 534.0**^B^** | 509-565 |
| TDS (mg L^-1^) | 476.8 | 434.0-505.0 | 472.3 | 441.0-529.0 | 437.2 | 298.0-524.0 | 445.2 | 370.0-525.0 | 414.1 | 334.0-491.0 | 390.8 | 344.0-442.0 |
| **^4^** TSS (mg L^-1^) | 17.4**^A^** | 4.9-51.3 | 7.5**^AB^** | 2.2-9.3 | 8.9**^AB^** | 4.2-11.2 | 5.8**^AB^** | 4.1-7.6 | 8.3**^AB^** | 5.7-11.4 | 4.3**^B^** | 2.5-6.0 |
| **^5^** ISS (mg L^-1^) | 7.6**^A^** | 0.4-27.8 | 1.5**^AB^** | 1.2-1.8 | 4.8**^AB^** | 1.4-9.1 | 3.5**^AB^** | 1.6-6.3 | 3.1**^AB^** | 1.3-5.1 | 0.9**^B^** | 0.2-2.0 |
| TOC (mg L^-1^) | 2.22 | 1.57-4.58 | 2.82 | 1.26-7.68 | 1.49 | 1.02-2.15 | 1.15 | 0.96-1.46 | 1.44 | 0.55-2.44 | 1.59 | 1.19-2.97 |
| Chl *a* (µg L^-1^) | 0.63 | 0.37-0.95 | 0.71 | 0.53-0.87 | 0.78 | 0.41-1.08 | 0.73 | 0.27-1.12 | 0.83 | 0.49-1.06 | 2.39 | 0.45-4.51 |
| **^6^** PO_4_^3-^ (mg L^-1^) | 0.024**^A^** | 0.008-0.038 | 0.014**^AB^** | 0.004-0.029 | 0.017**^AB^** | 0.003-0.026 | 0.012**^AB^** | 0.005-0.021 | 0.008**^B^** | 0.003-0.017 | 0.019**^AB^** | 0.016-0.025 |
| **^7^** TP (mg L^-1^) | 0.126**^A^** | 0.036-0.192 | 0.040**^B^** | 0.019-0.064 | 0.049**^B^** | 0.023-0.089 | 0.062**^B^** | 0.024-0.094 | 0.052**^B^** | 0.013-0.116 | 0.051**^B^** | 0.049-0.054 |
| TN (mg L^-1^) | 0.329 | 0.190-0.480 | 0.263 | 0.170-0.320 | 0.285 | 0.174-0.490 | 0.235 | 0.119-0.330 | 0.292 | 0.198-0.516 | 0.244 | 0.180-0.354 |
| **^8^** NO_3_^-^ (mg L^−1^) | 0.139**^AB^** | 0.038-0.205 | 0.177**^A^** | 0.132-0.226 | 0.155**^A^** | 0.147-0.220 | 0.171**^AB^** | 0.052-0.182 | 0.112**^AB^** | 0.066-0.178 | 0.050**^B^** | 0.019-0.064 |
| NH_4_^3-^ (mg L^−1^) | 0.116 | 0.077-0.188 | 0.064 | 0.022-0.086 | 0.071 | 0.048-0.086 | 0.059 | 0.019-0.086 | 0.031 | 0.015-0.043 | 0.042 | 0.029-0.069 |
| HCO_3_^-^ (mg L^-1^) | 226.6 | 200.2-244.1 | 230.5 | 213.6-246.5 | 233.2 | 212.8-251.4 | 211.9 | 198.9-230.7 | 206.7 | 179.4-231.9 | 219.8 | 205.0-236.8 |
| SO_4_^-^ (mg L^-1^) | 104.5 | 73.0-142.0 | 90.2 | 69.0-127.0 | 99.2 | 72.0-147.0 | 155.4 | 70.0-220.0 | 133.0 | 87.0-184.0 | 91.5 | 79.5-110.0 |
| **^9^** Ca^2+^ (mg L^-1^) | 99.4**^AB^** | 85.8-110.0 | 82.7**^AB^** | 27.5-101.2 | 99.6**^AB^** | 86.9-124.3 | 128.9**^A^** | 94.6-151.8 | 118.9**^A^** | 101.2-148.5 | 68.1**^B^** | 12.9-91.0 |
| **^10^** Cl^-^ (mg L^-1^) | 193.1**^A^** | 167.6-218.8 | 215.9**^A^** | 175.9-244.8 | 161.5**^A^** | 13.3-293.3 | 35.9**^AB^** | 12.8-114.8 | 14.2**^AB^** | 11.0-18.9 | 10.5**^B^** | 7.5-12.0 |
| Mg^2+^ (mg L^-1^) | 13.30 | 8.57-21.03 | 22.45 | 9.94-72.49 | 12.18 | 8.66-17.01 | 15.86 | 12.25-18.09 | 13.71 | 11.10-17.94 | 13.15 | 10.45-16.01 |
| **^11^** Na^+^ (mg L^-1^) | 59.26**^AB^** | 51.50-70.10 | 69.83**^A^** | 58.50-86.70 | 52.38**^AB^** | 4.60-95.20 | 12.62**^AB^** | 4.80-40.50 | 4.70**^B^** | 4.00-5.40 | 4.76**^B^** | 3.90-6.40 |
| K^+^ (mg L^-1^) | 1.95 | 1.80-2.10 | 2.00 | 1.70-2.31 | 1.85 | 1.32-2.11 | 1.92 | 1.44-3.61 | 1.48 | 1.20-1.90 | 1.52 | 1.20-2.10 |

**^1^** *H* = 22.96, *N* = 50, *df* = 5, *P* = 0.0003; **^2^** *H* = 23.68, *N* = 42, *df* = 5, *P* = 0.0003; **^3^** *H* = 20.34, *N* = 34, *df* = 5, *P* = 0.0011; **^4^** *H* = 11.49, *N* = 36, *df* = 5, *P* = 0.0325; **^5^** *H* = 12.78, *N* = 36, *df* = 5, *P* = 0.0255; **^6^** *H* = 15.92, *N* = 37, *df* = 5, *P* = 0.0071; **^7^** *H* = 14.36, *N* = 50, *df* = 5, *P* = 0.0135; **^8^** *H* = 18.33, *N* = 30, df=5, *P* = 0.0026; **^9^** *H* = 19.39, *N* = 33, *df* = 5, *P* = 0.0016; **^10^** *H* = 23.53, *N* = 34, *df* = 5, *P* = 0.0003; **^11^** *H* = 20.72, *N* = 33, *df* = 5, *P* = 0.0009;

**Supplementary Table S3**. The concentration of trace elements in the Kuźnica clarification pond in 2014-2019. Values with the different superscripts are significantly different among the years by non-parametric Kruskal–Wallis test (*p* < 0.05)

| Parameters | 2014 | | 2015 | | 2016 | | 2017 | | 2018 | | 2019 | |
| --- | --- | --- | --- | --- | --- | --- | --- | --- | --- | --- | --- | --- |
|  | Average | Range | Average | Range | Average | Range | Average | Range | Average | Range | Average | Range |
| *****Fe (mg L^-1^) | 3.84**^A^** | 2.03-5.80 | 2.00**^A^** | 1.81-2.15 | 2.80**^A^** | 2.43-3.42 | 1.45**^A^** | 1.29-1.65 | 1.19**^A^** | 0.97-1.40 | 0.61**^B^** | 0.32-1.01 |
| Mn (mg L^-1^) | 0.24 | 0.21-0.26 | 0.13 | 0.05-0.21 | 0.20 | 0.14-0.25 | 0.36 | 0.18-0.51 | 0.28 | 0.24-0.43 | 0.23 | 0.06-0.32 |
| Si (mg L^-1^) | 13.46 | 12.48-5.15 | 15.02 | 14.85-5.33 | 16.78 | 14.41-17.98 | 15.05 | 14.53-15.98 | 15.33 | 14.97-15.67 | 14.61 | 14.02-15.19 |
| Ag (µg L^-1^) | 0.01 | 0.01-0.02 | 0.01 | 0.01-0.02 | 0.01 | 0.01-0.02 | 0.01 | 0.01-0.02 | 0.02 | 0.01-0.02 | 0.01 | - |
| Al (µg L^-1^) | 1.97 | 1.85-2.14 | 2.01 | 1.14-2.58 | 2.38 | 1.42-2.87 | 2.35 | 1.95-2.97 | 2.25 | 2.04-2.48 | 2.02 | 1.97-2.07 |
| As (µg L^-1^) | 0.01 | 0.01-0.02 | 0.03 | 0.02-0.03 | 0.02 | 0.01-0.02 | 0.01 | 0.01-0.02 | 0.01 | 0.01-0.02 | 0.01 | - |
| Cd (µg L^-1^) | 0.01 | 0.01-0.02 | 0.01 | 0.01-0.02 | 0.01 | 0.01-0.02 | 0.01 | 0.01-0.02 | 0.01 | - | 0.01 | - |
| Cu (µg L^-1^) | 2.30 | 2.14-2.42 | 1.95 | 1.65-2.34 | 1.88 | 1.52-2.29 | 1.88 | 1.68-2.03 | 1.85 | 1.73-1.99 | 1.76 | 1.74-1.86 |
| Hg (µg L^-1^) | 0.01 | 0.01-0.02 | 0.01 | 0.01-0.02 | 0.01 | 0.01-0.02 | 0.01 | 0.01-0.02 | 0.01 | - | 0.01 | - |
| Ni (µg L^-1^) | 2.70 | 2.17-2.98 | 2.29 | 2.01-2.84 | 2.62 | 2.29-2.84 | 2.63 | 2.33-3.01 | 2.69 | 2.48-2.83 | 2.37 | 2.11-2.64 |
| Pb (µg L^-1^) | 0.93 | 0.75-1.06 | 0.89 | 0.65-1.04 | 0.64 | 0.42-0.87 | 0.68 | 0.53-0.84 | 0.67 | 0.60-0.73 | 0.56 | 0.51-0.62 |
| Se (µg L^-1^) | <0.01 | - | <0.01 | - | <0.01 | - | <0.01 | - | <0.01 | - | <0.01 | - |
| Zn (µg L^-1^) | 30.09 | 25.09-3.46 | 22.36 | 20.01-6.99 | 29.63 | 22.23-33.96 | 30.00 | 22.16-33.93 | 28.54 | 26.15-32.14 | 25.23 | 24.17-26.38 |

******H* = 18.12, *N* = 31, *df* = 5, *p* = 0.0028; - not defined

**Supplementary Table S4**. The values of partial metrics (MTB, MCB, MC) and final multimetric (PMPL) expressed as EQR, and ecological classification of the clarification pond in 2014-2019.

| Phytoplankton  metrics | **2014** | **2015** | **2016** | **2017** | **2018** | **2019** |
| --- | --- | --- | --- | --- | --- | --- |
| MTB | 1.00 | 1.00 | 0.97 | 1.00 | 0.96 | 1.00 |
| MCB | 0.98 | 0.98 | 0.98 | 0.98 | 0.98 | 0.98 |
| MC | 1.00 | 1.00 | 1.00 | 1.00 | 1.00 | 1.00 |
| PMPL | 0.996 | 0.996 | 0.985 | 0.996 | 0.982 | 0.996 |
| Ecological  classification^1^ | maximum | maximum | maximum | maximum | maximum | maximum |

MTB – metric „Total Biomass”, MCB – metric „Cyanobacteria Biomass” and MC – metric „Chlorophyll *a*”, PMPL – Phytoplankton Metric for Polish Lakes
